# Single beam echo-sounding dataset and digital elevation model of the southeastern part of the Baltic Sea (Russian sector)

**DOI:** 10.1016/j.dib.2019.104123

**Published:** 2019-06-11

**Authors:** Dmitry Dorokhov, Ivan Dudkov, Vadim Sivkov

**Affiliations:** Shirshov Institute of Oceanology, Russian Academy of Sciences, Russia

**Keywords:** Single beam echo-sounding, Digital elevation model, Relief, The Baltic Sea

## Abstract

We present the bathymetric dataset of a single beam sounding. Surveys were conducted in 25 expeditions of the Atlantic branch of Shirshov Institute of Oceanology RAS in the Russian EEZ area of the southeastern part of the Baltic Sea in the period from 2004 to 2018. Acoustic data were acquired by echo sounders Simrad EA-400SP and Furuno FS-700. The raw sounding data were filtered and corrected by sound velocity values. The dataset is presented as spreadsheets (*.xslx) and GIS point-class shapes (*.shp). The digital elevation model (DEM) of 1: 500 000 scale has been constructed for the entire Russian EEZ on base of the original array of sounding profiles and an open sources bathymetry [1]. DEM is presented by XYZ-grid (*.txt) and GeoTIFF raster (*.tif).

**Table:** Specifications table

Subject area	*Geology*
More specific subject area	*Sea geomorphology, bathymetry, hydrographic survey, oceanography*
Type of data	Spreadsheets (*.xslx), GIS feature classes (*.shp), Geotiff raster (*.tif), XYZ-grid (*.txt)
How data was acquired	*Continuous profiles of the bathymetric data acquired in marine surveys with using by the single-beam echo-sounders - Simrad EA 400 SP (Kongsberg Maritime, Norway) and Furuno FS-700 (Furuno, Japan).*
Data format	*Calibrated and processed spreadsheets, GIS layers, interpolated raster and XYZ-grid*
Experimental factors	The raw XYZ data of echo sounding (*.txt) were converted into MS Excel spreadsheets (*.xlsx). Data preprocessing included filtering and sorting. The spreadsheets were converted in the point shapefiles with depth marks. We digitized isobaths from*Ref. [1]**for the areas of insufficient data. The dataset also includes coastline zero marks. The bathymetric raster surface was calculated by linear interpolation.*
Experimental features	*Data include spatial point datasets suitable for analysis and mapping in GIS and interpolated raster of bathymetry*
Data source location	*Southeastern part of the Baltic Sea, EEZ of the Russian Federation*
Data accessibility	*Data are with this article*
Related research article	Sivkov V., Dorokhov D., Ulyanova M. Submerged holocene wave-cut cliffs in the South-eastern part of the Baltic Sea: reinterpretation based on recent bathymetrical data//J. Harff et al. (eds.) The Baltic Sea Basin. DOI 10.1007/978-3-642-17220-5_10, Springer-Verlag Berlin Heidelberg. - 2011. - pp. 203–217. ISBN: 978-3-642-17220-5

**Table:** 

**Value of the data** • The presented bathymetric dataset is unique for Russian sector of the southeastern part of the Baltic Sea. It contains the results of hydrographical works in 25 marine expeditions carried over the past 14 years. It was acquired by specialized hydrographic equipment and covers most of the study area. • High-resolution bathymetric profiles can be used for geomorphological studies of the bottom microrelief. • The bottom relief is the one of the main parameters of the bottom landscapes. The new DEM provides many opportunities for conducting geomorphological analysis and mathematical modelling of hydrological processes. • The presented raster DEM can be used as a cartographic basis for various thematic maps.

## 1 Data

**Fig. 1:**
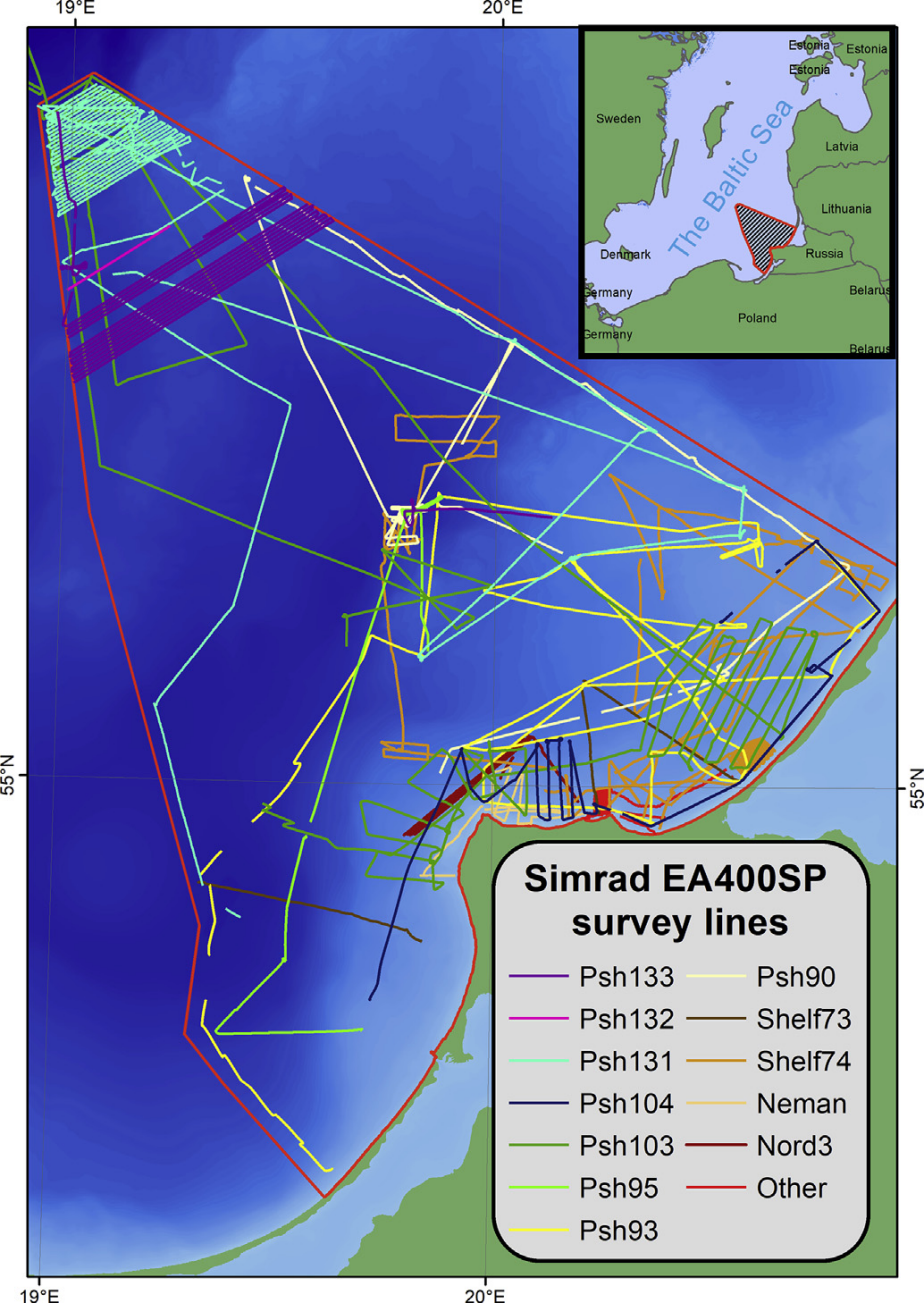
The single-beam echo-sounding profiles by Simrad EA400SP. Psh[NN] – cruise number by RV “Professor Shtokman”; Shelf[NN] – by RV “Shelf”; Neman, Nord3, RZH1-0810 – by small research vessels; NoNameBoat – by small rubber boats.

**Fig. 2:**
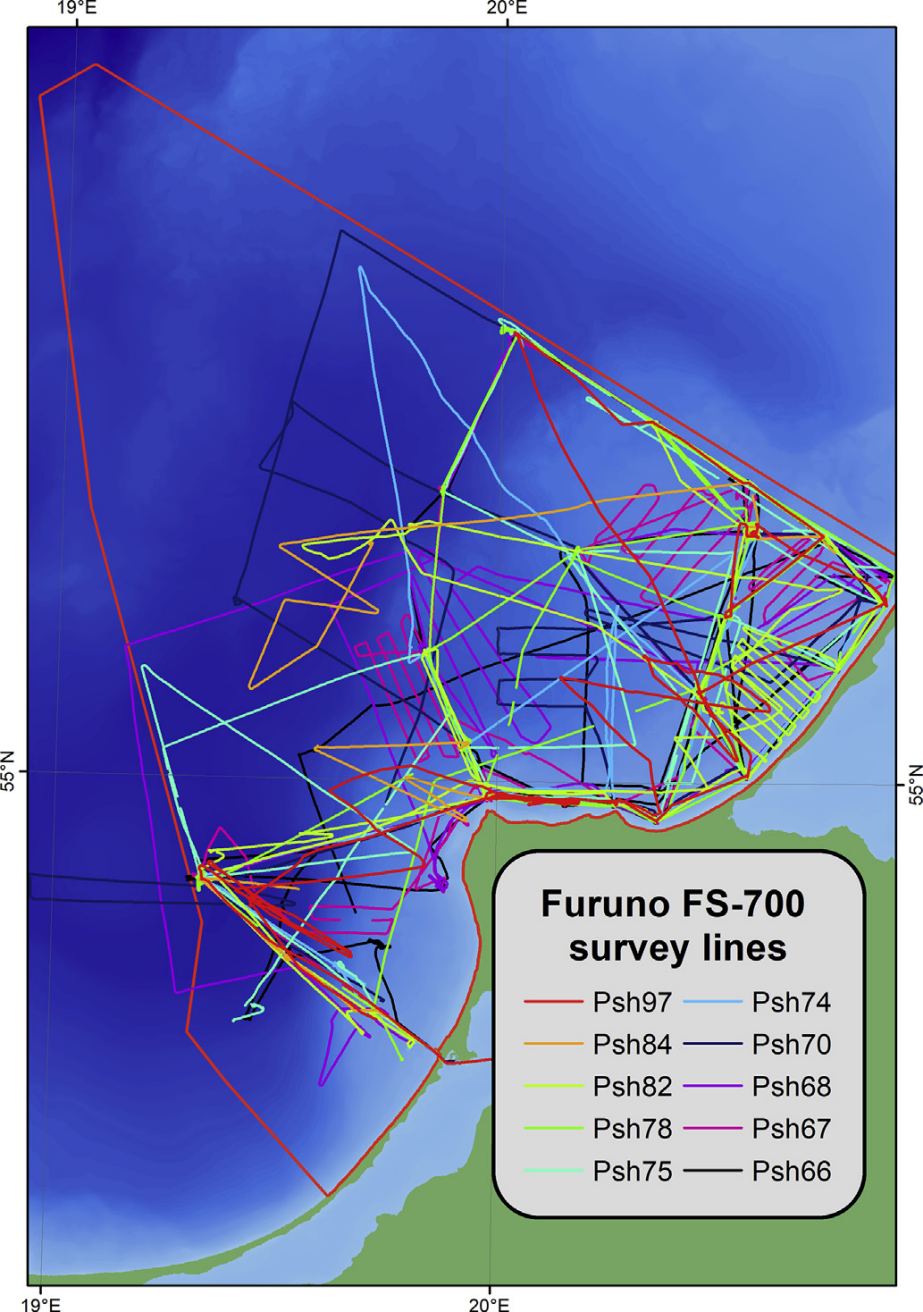
The single-beam echo-sounding profiles by Furuno FS-700, where Psh[NN] – cruise number by RV “Professor Shtokman”.
- The spreadsheets (*.xlsx) of thinned sounding data in WGS84 (distance between points is about 100–200 m);
- The ArcGis point class shapefiles (*.shp) of thinned sounding data in the UTM Zone 34 N projection, WGS84;
- The GeoTiff raster (*.tif) of DEM (Fig. 3) with a scale of 1:500 000 and spatial resolution of 200 m in the UTM Zone 34 N projection, WGS84;

The presented dataset includes:

- The spreadsheets (*.xlsx) of processed data of single-beam sounding obtained by Simrad EA400SP (Fig. 1) and Furuno FS-700 (Fig. 2) in WGS84;

**Fig. 3:**
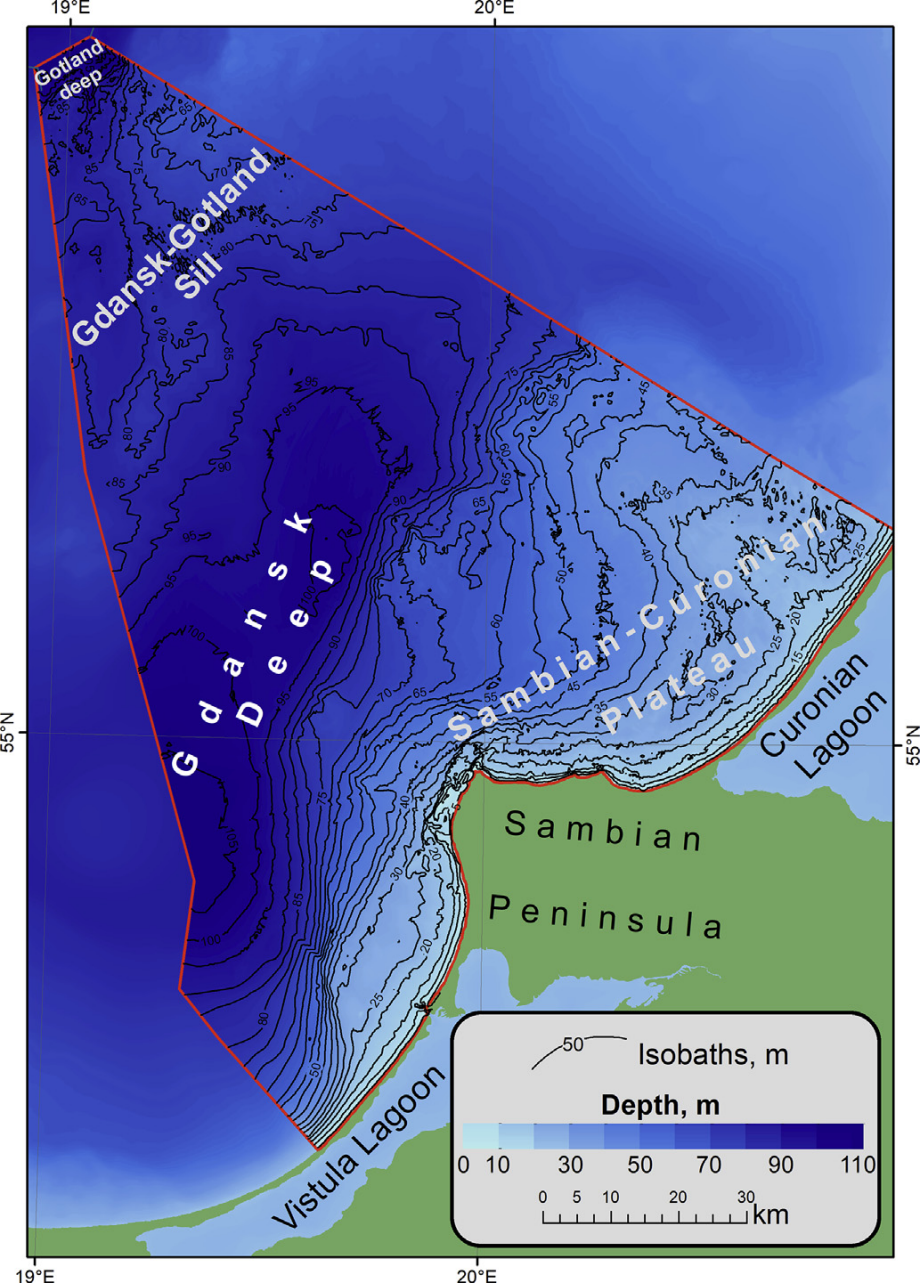
Bathymetric Map of Russian Part of the South-Eastern Baltic Sea on the base of presented data.
- The ASCII XYZ-grid (*.txt) of DEM values in the UTM Zone 34 N projection, WGS84;
- The ESRI ASCII Grid (*.txt) of DEM values in the UTM Zone 34N projection, WGS84;
- The isobaths in ArcGis lines feature class (.shp).

The structure of the spreadsheets (*.xlsx) and the attribute tables of the shapefiles (*.shp) is presented in Table 1.

**Table 1:** Description of columns in Excel spreadsheets and shapefiles attribute tables.

Column Name	Description
Vessel	Research vessel: Professor Shtokman (abbreviated - Psh), Neman, Shelf, RZH1-0810 and no-name small rubber boats.
Cruise_num	Number of the expedition cruise (for Psh and Shelf only).
Date	Date of survey, dd.mm.yyyy.
Longitude	Geographical longitude, decimal degrees, WGS1984.
Latitude	Geographical latitude, decimal degrees, WGS1984.
Device	Echo sounder device: Furuno FS-700 or Kongsberg Simrad EA 400SP.
Channel	Survey frequency channel: 50, 38 or 200 kHz.
Raw_depth	Raw depth, m. Corr_in_console – corrected in device console during of sounding.
SV_corr	Sound velocity profile correction, m. Corr_in_console – corrected in device console during of sounding.
Depth	Processed depth, m = Raw_depth (m) + SV_corr(m) + Transd_d(m).
Transd_d	Depth of transducer submergence, m.

## 2 Experimental design, materials, and methods

### 2.1 Study area and expeditions

The study area is the Russian EEZ and territorial waters in the southeastern part of the Baltic Sea (Fig. 3).

The bottom relief of the Baltic Sea is characterized by series of depressions and sills between them, narrow troughs and shallow banks. The Gdansk basin is the most part of the study area. There is the Gdansk-Gotland Sill on the north of the study area. It separates the Gdansk Deep from the Gotland Deep (Fig. 3). The Gdansk Deep is a large negative relief form of the Gdansk Basin with maximum depth of about 110 m. There is a large positive form of relief – the Sambian-Curonian Plateau in the eastern part of the Gdansk basin.

**Fig. 4:**
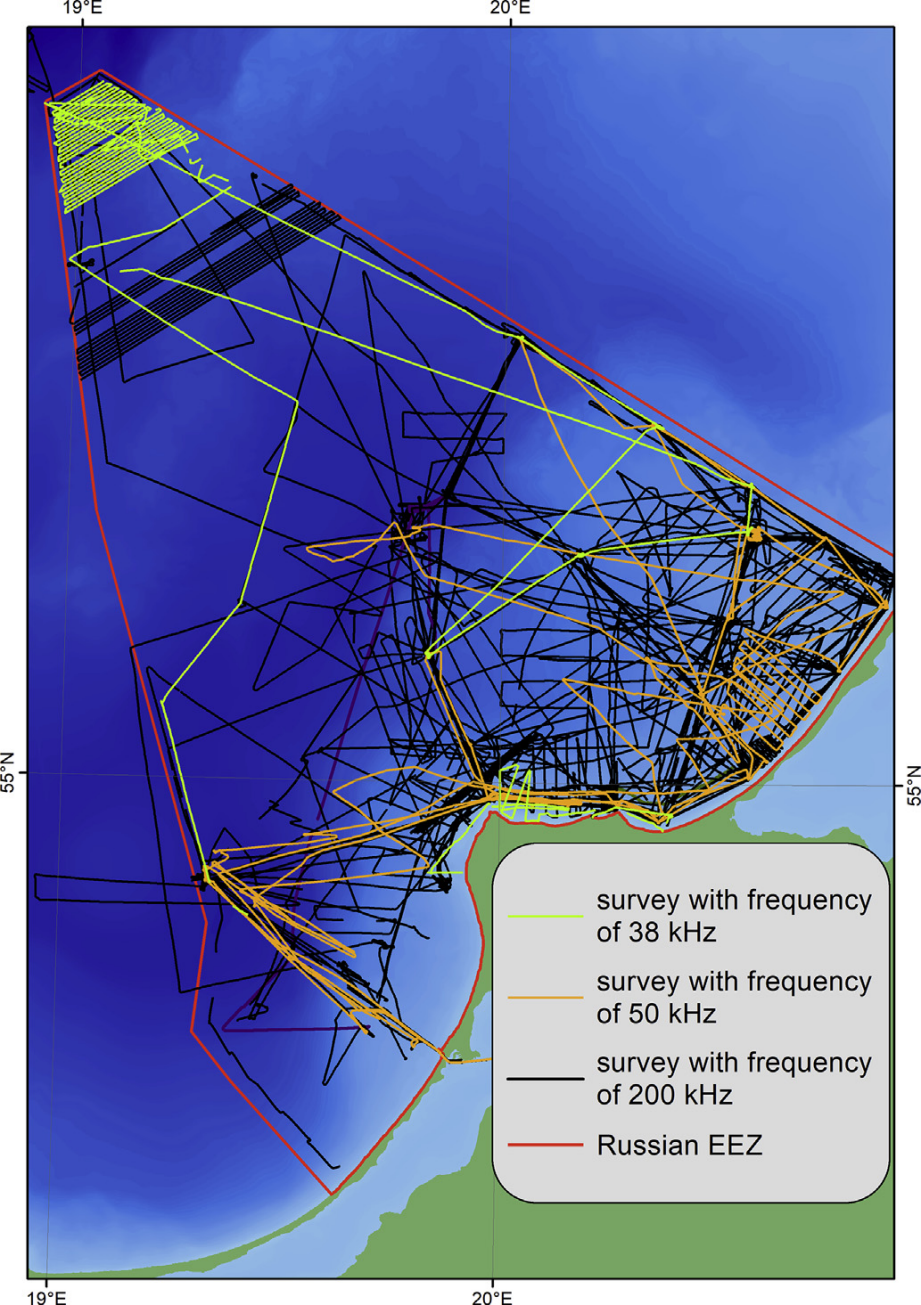
Echo-sounding tracks divided by survey frequency channels.

The expeditions were carried out by the Atlantic Branch of the Shirshov Institute of Oceanology of the Russian Academy of Sciences with use of various research vessels, mostly on RV “Professor Stockman”. The map of original sounding data is on Fig. 4. The soundings were acquired by narrow-beam high-frequency echo-sounder Simrad EA400SP (Kongsberg, Norway) (Fig. 1) and the ship-installed echo-sounder Furuno FS-700 (Fig. 2).

The Simrad EA400SP is hydrographic two-frequency echo-sounder operated at 38 and 200 kHz frequency channels. As a rule, we used the frequency of 200 kHz because it gives more exact depth then lower frequencies. We used the frequency of 38 kHz as on dense sediments and with the absence of the technical possibility of using the frequency of 200 kHz (Fig. 4). The accuracy of depth measurements is 1 cm at 200 kHz frequency and 5 cm at 38 kHz.

The echo-sounder Furuno FS-700 was operated at 50 and 200 kHz frequency channels (Fig. 4). It was used in the absence of the Simrad EA400SP on a board. The accuracy of the Furuno FS-700 is 0.1 m or 2% of the depth.

The measured depths and coordinates from DGPS were recorded in the echo-sounders console in the digital format. The depths were measured from the echo-sounder transducer. The echograms and depth values were recorded in *.raw format which was converted into XYZ tables (*.txt) for further processing. We measured the temperature and salinity profiles for the sound velocity parameters (SVP) corrections in each expedition.

### 2.2 Processing of the sounding data

The processing of raw data was performed in the MS Excel software. The raw data were converted from the XYZ data files using macros written in the Visual Basic for Applications (VBA). The data were filtered for outliers and spurious depth values. The SVP and position of the transducer below water level were used for the depths correction. The processed data were compounded in spreadsheets (*.xlsx) as shown in Table 1. These data were thinned through 150–200 m using a VBA macros and saved in another spreadsheets (*.xlsx). The spreadsheets of thinned data were converted into ArcGIS in point-class shapefiles (*.shp) of WGS1984 UTM Zone 34 N projection for further use and construction of the DEM.

### 2.3 The DEM construction

The dataset for DEM construction includes:

- the echo sounding data presented in this article;
- the digitized isobaths of the bathymetric map of the Central Baltic Sea [1];
- the digitized isobaths of the coastal nautical charts.

**Fig. 5:**
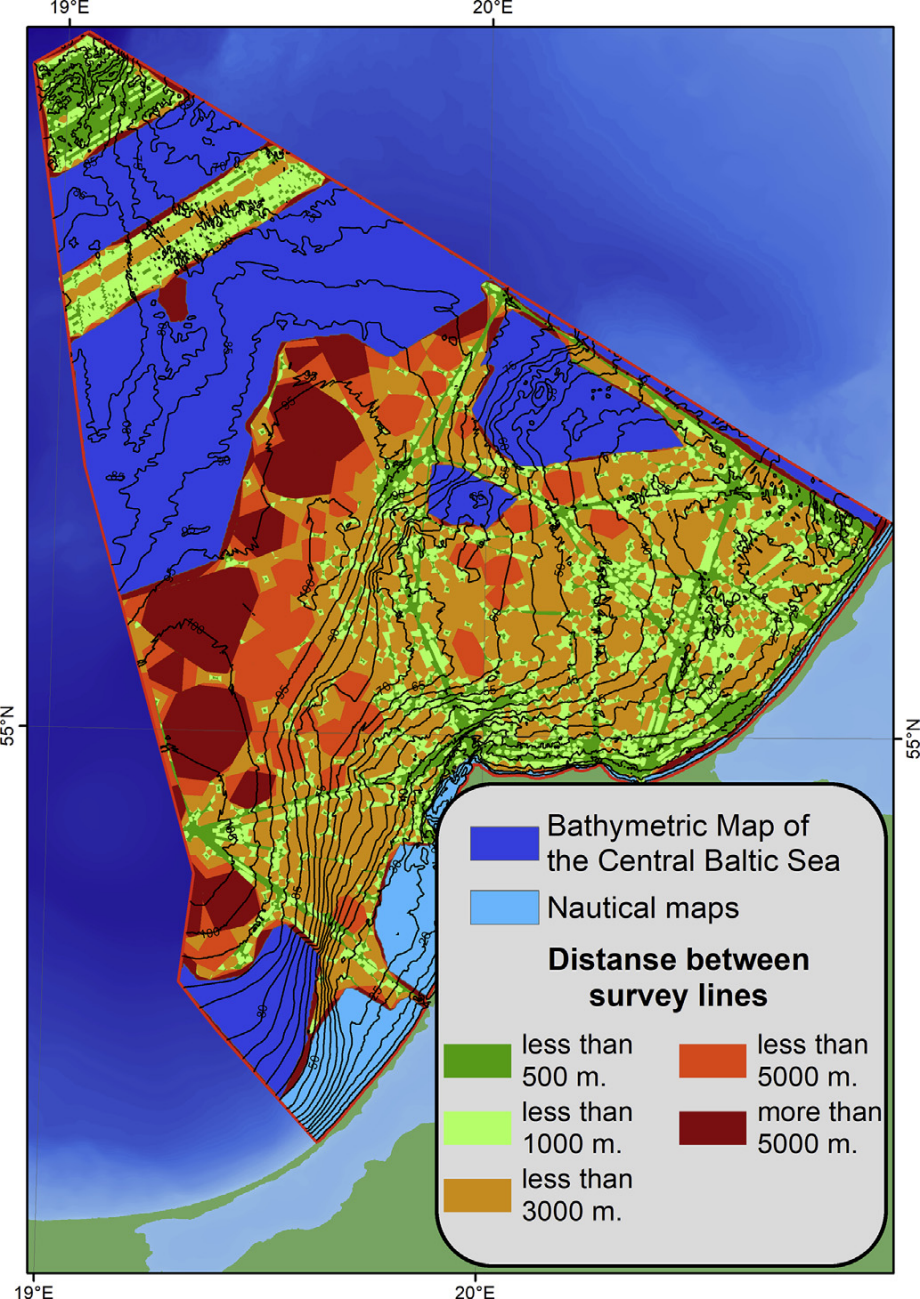
Map of used data for the DEM construction.

The shapefiles of the sounding data were plotted in ArcGIS. The areas with insufficient data were determined (Fig. 5). Isobaths from Ref. [1] and nautical charts were digitized and net of regular depths were calculated for these areas. These depth values were converted to the point shapefiles. We also added coastline zero marks as point shapefile to the general array for correct interpolation. All shapefiles of general data array were combined into ArcGIS dataset. From that one we created a terrain dataset by using the ArcGIS 3D Analyst module (Terrain To Raster). In this instrument, natural neighbor interpolation is applied through the triangulated terrain surface. The DEM has a spatial resolution of 50 m and a horizontal scale of 1:500 000 (Fig. 3). The obtained DEM of the bottom relief is presented as the GeoTIFF raster (*.tif), ASCII XYZ-grid (*.txt) and the ESRI ASCII Grid (*.txt).
