# Supplementary material for: Single beam echo-sounding dataset and digital elevation model of the southeastern part of the Baltic Sea (Russian sector)
Source: Data Brief. 2019 Jun 11;25:104123. doi: 10.1016/j.dib.2019.104123 (PMC6598838; doi:10.1016/j.dib.2019.104123)
Supplement: Multimedia component 5 [file mmc5.docx]

Folder tree:

1. Folder “Kongsberg Simrad EA-400SP”
   1. Folder “VesselNameNum_Frequency”
      1. Folder “shp_sp” - GIS points feature class (.shp)
      2. Folder ”xlsx_pp” – processed data
         1. Vessel_Cruise number_Device_ Frequency.xlsx
      3. Folder “xlsx_pp_thin” – thinned data
         1. Vessel_Cruise number_Device_ Frequency.xlsx
2. Folder ”Furuno FS-700”
   1. Folder “VesselNameNum_ Frequency”
      1. Folder “shp_sp” - GIS points feature class (.shp)
      2. Folder ”xlsx_pp” –processed data
         1. Vessel_Cruise number_Device_ Frequency.xlsx
      3. Folder “xlsx_pp_thin” –thinned data
         1. Vessel_Cruise number_Device_ Frequency.xlsx
3. Folder “Geo Tiff” – GeoTiff bathymetry raster
4. Folder “DEM_XYZ” – bathymetry in XYZ ASCII format
5. Folder “DEM_ASCII” – bathymetry in ESRI Grid ASCII format
6. Folder “Isobaths” – isobaths in GIS lines feature class (.shp)
